# Exploring Emotional and Cognitive Perceptions of Cervical Cancer Screening and Self‐Sampling Among Vulnerable Romanian Women: A Qualitative Study

**DOI:** 10.1111/hex.70485

**Published:** 2025-11-30

**Authors:** Nicoleta‐Monica Pașca, Diana Tăut, Rachel Greenley, Pia Kirkegaard, Rikke Buus Bøje, Martin McKee, Marc Bardou, Adriana‐Smaranda Băban, and the CBIG‐SCREEN Consortium

**Affiliations:** ^1^ Department of Psychology Babeș‐Bolyai University Cluj‐Napoca Romania; ^2^ London School of Hygiene and Tropical Medicine, Centre for Global Mental Health London UK; ^3^ University Research Clinic for Cancer Screening, Randers Regional Hospital Randers Denmark; ^4^ Department of Clinical Medicine Aarhus University Aarhus Denmark; ^5^ Department of Health Services Research and Policy, London School of Hygiene and Tropical Medicine London UK; ^6^ Inserm‐Centre d'Investigations Cliniques 1432 (CIC 1432), CHU Dijon‐Bourgogne Dijon France

**Keywords:** cervical cancer, cervical cancer screening, prevention, vulnerable population

## Abstract

**Introduction:**

This study aimed to explore the emotional and cognitive perceptions of cervical cancer screening (CCS), with a focus on self‐sampling for human papillomavirus (HPV) by vulnerable Romanian women.

**Methodology:**

Eighteen semi‐structured individual interviews were conducted with vulnerable women, defined by their low socioeconomic status (SES), residence in rural areas and membership in ethnic minorities. Participants were recruited using snowball sampling. Interviews followed a guide designed to assess constructs from the Health Belief Model (knowledge, barriers, evaluation of vulnerability) as they relate to women's views of prevention, the healthcare system and CCS. A Think Aloud protocol was used with a subsample of six women to identify potential barriers, opportunities and attitudes related to the use of self‐sampling kits. A reflexive thematic analysis was conducted to identify the primary themes related to with women's perceptions of CCS and self‐sampling.

**Results:**

Four major themes emerged: (a) risk, fear and other emotional experiences; (b) women's perception of barriers; (c) knowledge, personal control and cues to action; and (d) women's perception of self‐sampling. Participants unravelled emotional and logistical barriers to CCS and self‐sampling. Limited health literacy, fear and shame influenced risk perceptions, hindering CCS participation. Logistical challenges and financial constraints further impeded access, while women often prioritized the needs of their families over their own health. The Think Aloud protocol revealed that initial concerns regarding self‐sampling could be alleviated by having clear instructions, highlighting the importance of support during the decision‐making process.

**Conclusion:**

This study identified diverse barriers preventing vulnerable Romanian women from engaging in CCS, including logistical challenges, financial constraints and emotional factors.

**Patient or Public Contribution:**

Patients or the public were not involved in the design, conduct, analysis or interpretation of the study.

**Abbreviations**
CC: cervical cancer
CCS: cervical cancer screening
GP: general practitioner
HBM: Health Belief Model
HCPs: healthcare professionals
HPV: human papillomavirus
SES: socioeconomic status
STDs: sexually transmitted diseases

## 1 Introduction

Cervical cancer (CC) is the fourth most common neoplasia among women worldwide in terms of its incidence and mortality [1]. Human papillomavirus (HPV) vaccination combined with cervical cancer screening (CCS) is highly effective in reducing CC's morbidity and mortality. However, the implementation of primary and secondary prophylaxis varies greatly. Moreover, addressability in the targeted population is modest, as revealed by a low CCS coverage within the previous 5 years of around 32% globally, among women aged 30–49 [2]. Socioeconomic disparities significantly contribute to the global uptake gap. One review reported coverage in high‐income countries (HICs), reaching up to 77%, in the target population, but 9%–24% in low‐ and middle‐income countries (LMICs) [2]. Western and Central European countries report CCS coverage above 70%, while Eastern European countries report the lowest rates, at 35%–49%. Romania's CCS coverage is under 30%, with an infection rate of 12.2–15.4 per 100,000 women [3]. The country has the highest incidence and mortality attributed to CC in Europe [4, 5]. Quintal and Antunes reported that 62.2% of Romanian women had never been screened [6]. Although the country launched a national CCS programme in 2012, offering free cytology tests every 5 years for women aged 25–64, it lacks an active invitation system or linkage to a population registry [7]. The lack of an organized programme poses a major barrier to women, particularly for vulnerable ones [8]. Despite concerted policy efforts to improve uptake, understanding the views of women who remain unscreened requires a more granular appreciation of the barriers they face.

Vulnerable women are at risk of adverse health outcomes for several reasons. These include living in poor, violent, isolated or socially deprived areas; having sexually transmitted diseases (STDs); being immigrants or belonging to ethnic minorities (e.g., Roma); being unemployed; lacking health insurance; having little or no formal education; struggling with limited economic resources or experiencing homelessness [9, 10]. Emotional and knowledge‐based barriers, such as shame and embarrassment regarding gynaecological examinations, internalized stigma, distrust in the healthcare system or healthcare professionals (HCPs) and lack of awareness or information about CC and/or CCS, deepen the problem. Practical obstacles include transportation issues, competing demands and limited access to healthcare [9, 11, 12, 13, 14, 15, 16]. Additionally, limited health literacy can discourage CCS participation and raise concerns about the procedure and any follow‐up care [17]. All these systemic and individual barriers combine synergistically, impeding their adherence to standardized screening procedures, with adverse health consequences. In Romania, almost half of the population resides in rural areas, one of the highest percentages in Europe. Moreover, more than 20% of the population battles poverty, and more than 10% are members of ethnic minorities [18, 19]. Therefore, a significant portion of the population might be considered vulnerable and at risk for adverse health behaviours and low adherence to screening.

Several strategies have been tested to improve adherence to CCS, with self‐sampling emerging as one of the most promising approaches. This method involves collecting cervicovaginal cells using swabs, brushes, tampons or lavage devices [20]. A growing number of European countries, including Denmark, Finland, France, Sweden, Greece and Portugal, have explored self‐sampling for HPV testing, particularly among vulnerable women. It has gained attention as a way to reach women who are overdue for screening, with kits often sent by mail alongside reminder letters [21]. Some countries, such as Albania, the Netherlands, Italy and Spain, have already implemented self‐sampling as a standard option for all women [22]. A systematic review evaluating women's preferences for self‐sampling versus clinician‐collected samples yielded mixed results. However, it identified intimacy and convenience as key advantages of, with women being twice as likely to choose self‐sampling over standard care [23]. These benefits make self‐sampling especially valuable for increasing coverage and accessibility in hard‐to‐reach or underserved populations. When combined with HPV vaccination and traditional cytology, self‐sampling could help close screening gaps and reduce CC‐related morbidity and mortality [19].

Despite growing interest in self‐sampling as a strategy to improve CCS, research on its acceptability and the underlying barriers among Romanian women, particularly those in underserved communities, remains limited. Vulnerable women face complex, multidimensional deterrents to CCS and the follow‐up of abnormal results may further heighten scepticism and fear towards testing [24]. Additionally, self‐sampling introduces further logistical challenges, especially regarding continuity of care and follow‐up procedures, which can be particularly difficult for disadvantaged populations [25]. These factors contribute to both intrinsic and systemic barriers to screening. While self‐sampling holds promise for improving outcomes, its implementation must overcome these challenges. Therefore, its acceptability and feasibility should be thoroughly investigated, especially in countries and regions where such approaches are not yet established [9, 11, 12, 13, 14]. Future research should explore self‐sampling in diverse social, cultural and national contexts, as few studies have examined the specific barriers to CC prevention among at‐risk Romanian women.

This study, conducted as part of the CBIG‐SCREEN Project (Co‐creating the Offer of Cervical Screening with Vulnerable Groups for Vulnerable Groups), aimed to reduce inequalities in CCS among vulnerable populations. To achieve this, a contextualized approach was adopted, one that considered country‐specific prevention policies and cultural characteristics, to identify feasible solutions to existing barriers. The study focused on exploring the emotional and cognitive perceptions of CCS, with particular attention to self‐sampling for HPV among vulnerable Romanian women. By doing so, it provided a more nuanced understanding of the multifaceted barriers these women face in accessing screening services.

## 2 Methodology

### 2.1 Participants

Semi‐structured individual interviews (*N* = 18) were conducted with vulnerable Romanian women (characterized by low socioeconomic status (SES), living in rural or remote areas and belonging to ethnic minorities) between December 2022 and March 2023. An additional Think Aloud protocol was implemented in a subsample of vulnerable women to explore the acceptability of self‐sampling procedures (*N* = 6).

### 2.2 Sampling

Participants were recruited using the snowball sampling, a non‐probabilistic method in which initial participants refer others from their community. Initial contacts were made through a local health mediator involved in health promotion campaigns in underserved, rural, isolated and predominantly ethnic minority communities. These referrals led to the inclusion of women from similar backgrounds. Six interviews were conducted in person in Cluj County, where investigators were based, in a rural area where most inhabitants were ethnic minorities (Roma population). These interviews were arranged with the support of the health mediator. The remaining interviews were conducted by phone, either due to geographic distance, participant preference or limited accessibility. The combination of face‐to‐face and telephone interviews was chosen to maximize reach among isolated and sceptical respondents, while also allowing the Think Aloud protocol to be conducted in person where feasible.

### 2.3 Consent Procedures

Before participating, women were informed about the study's purpose and provided written or verbal informed consent (for respondents contacted by phone and those with limited reading and/or writing abilities). Two trained interviewers conducted the interviews, either in person or by telephone. For those who consented, the Think Aloud protocol was conducted face‐to‐face following the in‐person interviews. Interviews lasted between 20 and 70 min. The Ethical Committee of Babes‐Bolyai University Cluj‐Napoca approved this study, Reference Number 557/29.11.2022.

### 2.4 Instruments

The interview guide was developed by a transnational team within the CBIG‐SCREEN Consortium, starting from the determinants of health behaviours as described in the Health Belief Model (HBM) [26]. The HBM is a valuable framework for inquiring about feelings related to personal risk, costs and benefits of CCS, self‐efficacy, knowledge and expectations [27, 28, 29]. The framework was adapted to include questions about participants' feelings and experiences with the healthcare system and their general, obstetrical and gynaecological care.

The Think Aloud protocol addressed the problem‐solving process while capturing participants' thoughts about navigating a task and sharing them with the interviewer [30]. The Think Aloud was applied to a subsample of six women (all interviewed face‐to‐face). Participants were given a self‐sampling kit and instructed to unwrap it and describe verbatim their opinion as they inspected the probe, read the instructions and imagined completing the procedure at home. Interviewers provided gentle prompts when participants struggled to articulate their thoughts or expressed uncertainty. The complete interview guide and Think Aloud protocol are available in Supporting Material S1.

### 2.5 Data Analysis

Reflexive thematic analysis was employed to interpret the data, and to explore and develop patterns of meaning in the participants' narratives [31]. All interviews were audio‐recorded with the participants' consent, then transcribed and analyzed in Romanian. No software was used for the qualitative analysis. An inductive and deductive thematic analysis was applied to derive codes, which were subsequently merged into sub‐themes and themes. Relevant keywords and sentences were identified after comprehensive readings of which codes were based on. After the codes were derived, emerging patterns were merged into principal themes. The coding was carried out manually, by two independent researchers (D. T. and N. M. P.), following the areas outlined in the interview guide and accommodating new themes that emerged from the interviews. Discrepancies between coders were reviewed and discussed and finally settled by a third investigator. For the Think Aloud data, inductive thematic analysis was conducted by two independent researchers (D. T. and R. G.) and settled by a third, when needed. The Think Aloud method nuanced the problem‐solving process regarding self‐sampling and was incorporated into the interview themes and sub‐themes [30].

## 3 Results

Most participants (15/18) resided in rural areas of Cluj and Maramureș counties and had low SES. The majority were unemployed, with one‐third lacking health insurance. Most were married, aged between 25 and 60 (*M* = 42.33, SD = 11.69) and had between 0 and 9 children (see Table 1).

**Table 1:** Population characteristics.

Variable		Number
Age	20–30 years old	4
	31–40 years old	5
	41–50 years old	4
	51–60 years old	5
Education	No school	1
	Elementary	1
	Primary school	0
	Secondary school	4
	Higher	6
	No information	6
Number of children	No children	2
	One child	7
	Two children	4
	Three children	3
	More than three children	2
Type of vulnerability	Unemployed	12
	Ethnic minorities	2
	Low socioeconomic status	15
	Living in rural or remote areas	15
Health insurance status	Uninsured	6
	Insured	11
	Co‐insured^a^	1

a Status of insurance for people with no income but who have a relative (parent, spouse, child) with health insurance that chooses to co‐insure them.

### 3.1 Risk, Fear and Other Emotional Experiences

#### 3.1.1 Underestimation of Personal Risk

Risk appraisals refer to the individual's perceived probability of developing a disease such as CC. Women's perceptions of their risk for developing CC varied from low to high.

I think I have a high risk. […] Now many people are diagnosed with cancer and with CC. I do not know what is happening.

Interview 15, 45 years old – rural area

Most participants struggled to reflect on their own risk of developing CC. This difficulty carried negative emotional undertones, such as fear or dread of cancer. One participant did not engage in this ‘*risk conversation*’ because of the cancer dread it aroused. Thinking about risk was seen as a bad omen that could be blocked by saying, ‘*God forbid!*’ *(Interview 14, 39 years old, rural area)*. In this respect, divinity was another reason for their risk evaluations: God wouldn't do that to them, and their confidence in divinity would mitigate the risk.

I don't know how to explain it. That's how I go with this belief that I have a small chance (to develop CC). I trust that I won't. I trust God that God won't let me have that disease, cancer. He takes care of me, so I don't get that disease.

Interview 5, 37 years old, low SES

Age was also a crucial factor for the low‐risk evaluation:

I would say that, now, I have a low risk because I am not that young anymore.

Interview 8, 58 years old, rural area

#### 3.1.2 Fear, Guilt and Empowerment: Emotional Dimensions of CCS

The prospect of undergoing CCS triggered significant apprehension among participants. Many expressed the dread of receiving a potentially fatal diagnosis, compounded by anxiety about lacking solutions for the future, particularly regarding the well‐being of their children. The fear of a cancer diagnosis and the hurdles of accessing healthcare, such as the absence of insurance and financial constraints, contribute to overwhelming feelings of helplessness and hopelessness. Some women grappled with guilt over their inaction, possibly rooted in a perceived inability to navigate the healthcare system. For some women, avoiding screening altogether became a coping mechanism, offering temporary comfort and cultivating a sense of hope for good health despite the uncertainty. However, for these women, the idea of CCS was closely associated with increased anxiety and anticipatory regret, especially in the absence of accessible treatment options. Ultimately, the fear of a grim diagnosis and the perceived inability to cope served as a major deterrent to screening participation.

I would not have a problem (attending CCS) if I did not have children: […] if the doctor gave me three months to live,[…] how do you tell your child that? “I didn't take care of myself, and now I am dying.” […] It is excruciating […] And the fear that I cannot treat myself… and maybe I've had it (CC) for many years. If I do not go, I do not know. […] and stress kills you faster than the disease!

Interview 1, 42 years old, uninsured

On the contrary, for some women, the fear of a threatening diagnosis serves as a motivating force, driving them to embrace CCS as a personal responsibility. Empowered by the prospect of preventive measures, women perceive CCS as an opportunity to safeguard their health proactively. However, the decision becomes more stressful, especially if symptoms are present, heightening anxieties about potential diagnoses.

I can't lie that I'd be a bit scared, but I would. At least I'd know what was going on. You can prevent it. If you do it, do it timely so you can really prevent it. If you stay until the last minute, you might not be able to do anything with it.

Interview 4, 30 years old, uninsured

#### 3.1.3 Navigating Shame and Embarrassment in CCS

The age factor significantly impacts women when considering gynaecological examinations that involve body exposure. Feelings of shame and embarrassment, intertwined with societal perceptions of their bodies not aligning with youthful ideals, manifest as negative self‐images that pose potential barriers to women undergoing CCS.

After a certain age, you think: Why should I undress there? We wouldn't be so ashamed if we were the younger ones. And that keeps pulling you back.

Interview 17, 60 years old, rural areas

Considering the cultural context and the stigma surrounding an oncological diagnosis, the intense feelings of shame regarding one's health and the embarrassment associated with visiting the doctor are recognized as experiences that should not be imposed upon anyone. A participant succinctly encapsulates this: ‘*(not attending CCS) because of shame! Of course, because of that! But it should not be shameful because it is for your health.*’ *(Interview 17, 60 years old, rural areas)*. This insight underscores the urgent need to address and reshape societal norms and cultural perceptions around women's sexual health to alleviate the emotional burden of shame and embarrassment that impedes women from prioritizing their health through essential screenings.

### 3.2 Women's Perception of Barriers

#### 3.2.1 The Burden of Not Having Health Insurance

Insufficient financial resources compelled women to reconsider their priorities, invariably placing their children's immediate needs above their own health concerns. Financial constraints led to a reprioritization, with daily necessities like meals taking precedence over personal health. This lack of access to subsidized medical services increased the risk of self‐medication and led to the neglect of pressing health issues. The dilemma is palpable in one participant's reflection:

Should I go to the hospital in Cluj? However, my child is hungry; […] If I must pay, I'd better not go. I can go to the pharmacy instead, take painkillers, and my pain disappears.

Interview 1, 42 years old, uninsured

This choice underscores the intricate interplay between financial strain, the lack of insurance and women's difficult decisions in balancing their family's immediate needs with their own healthcare.

#### 3.2.2 Navigating the Referral System: Perceptions and Challenges

Although the referral system, intended as standard of care, many women described it as a confusing and burdensome journey. They must navigate a maze of HCPs, enduring long waiting times, and investing significant effort often left participants feeling frustrated and discouraged. Past negative experiences further shaped their perceptions, several women describing medical staff as indifferent and disengaged. Receiving a referral after minimal interaction was perceived as dismissive, reinforcing the belief that HCPs prioritize efficiency over meaningful connection. This perception contributed to a sense of disempowerment, discouraging women from actively seek care, as they felt that practitioners evade responsibility and hinder effective communication. One woman summarized this sentiment by stating:

If we go to a family doctor (general practitioner, GP), they do not even look at us or perform any physical exams. They ask me: What hurts? (I answer:) It hurts here. (The doctor responds:) Ok, I will give you a referral.

Interview 2, 40 years old, uninsured

One participant offered a particularly insightful perspective, viewing the referral system not merely as a bureaucratic process but a marker of SES. For her, receiving a referral symbolized financial limitations, a barrier that prevented access to opportunistic screenings available through private healthcare, which many others could afford. This perception was accompanied by feelings of humiliation, as private hospitals often prioritized individuals who could pay upfront or offer informal incentives, in stark contrast to the public system, where long waiting times and lower prioritization were common for those without immediate financial means. This narrative unveils the layered challenges women face within the referral system, encompassing both practical obstacles and deeper socioeconomic implications that contribute to a sense of disempowerment among vulnerable groups.

Because of being poor, I went to the public hospital with a referral note from the GP. […] You can also go with a referral note to a private practice. So, I went there and took a monthly referral when he (the gynaecologist) called me in. […] However, he (the gynaecologist) first let in the women who paid upfront. I had to stay until I could not stand it anymore while the ladies who gave money or bribes were prioritized.

Interview 1, 42 years old, uninsured

### 3.3 Knowledge, Personal Control and Cues to Action

#### 3.3.1 No Symptoms—No Screening

A prevalent theme among participants was a tendency to seek healthcare only when symptoms appeared, rather than engaging in preventive behaviours. This symptom‐driven approach was exemplified by one woman who asked:

What information, other than if something hurts down there (genital area), should prompt me to seek medical advice?

Interview 17, 60 years old, rural areas

Intriguingly, a notable subgroup of women challenged the idea that healthcare should only be sought when symptoms appear. They emphasized the indispensability of preventive services and regular check‐ups for early detection and proactive health management. While some women demonstrated awareness of preventive practices, they acknowledged that symptoms remained their primary motivator for action. Nonetheless, they recognized that taking personal responsibility for one's health and scheduling routine screenings are essential components of effective preventive medicine. One participant eloquently captured this perspective:

They (people) should take an interest in their lives, in themselves, and go and have their tests done to see what is going on. That is what is right. It is up to us. So, if I have common sense and want to go, I will go. I do not go if someone says so. I am not going because I am afraid […].

Interview 5, 37 years old, low SES

#### 3.3.2 Empowerment Catalysts: Family and HCPs

The catalysts for empowerment in CCS extend beyond individual decision‐making, encompassing the crucial roles of family support and HCPs. Participants emphasized the pivotal influence of familial encouragement, noting that a supportive family instils a sense of security and acts as a powerful motivator for those grappling with fear or uncertainties about the decision to undergo screening. One participant expressed:

The family should encourage you and say, go, do it; you'll be fine. Even if the outcome isn't what you expected. […] I would encourage them if I was their family.

Interview 4, 30 years old, uninsured

Furthermore, conversations with HCPs emerged as another empowerment vector. Participants revealed that speaking with specialists instilled a different kind of trust, regardless of the screening outcome: ‘*(talking to a specialist) gives you some courage and confidence.*’ *(Interview 4, 30 years old, uninsured)*. This newfound courage, bolstered by professional guidance, is a compelling factor in motivating women to actively embrace early prevention strategies such as CCS.

#### 3.3.3 Bridging Knowledge Gaps: Empowering Through Education and Awareness in Remote Contexts

The intersection of geographical isolation, limited education and low awareness levels unveils significant challenges in empowering women to participate in CCS. Participants emphasized that individuals residing in rural areas often meant restricted access to healthcare services. Isolation and lower levels of formal education contributed to a lack of understanding about CC and the importance of screening. Participants candidly expressed the challenges of rural life, with one remarking:

We (countrywomen) are also more closed‐minded, and we are not as smart as the city people.

Interview 1, 42 years old, uninsured

This sentiment reflects the educational disparities that frequently characterize rural communities. Another participant bluntly attributed her lack of awareness directly to limited schooling, stating:

I do not know anything (about CCS) […] because I do not have any schooling.

Interview 5, 37 years old, low SES

### 3.4 Women's Perception of Self‐Sampling

The Think Aloud protocol was used with the subsample of women interviewed in person. Women's journey with self‐sampling unfolds through the lens of curiosity, fear, knowledge acquisition and support‐seeking. Despite ongoing uncertainties persist, explicit instructions, emotional reassurance and perceived benefits play pivotal roles in shaping their willingness to consider self‐sampling as a viable method for CCS.

#### 3.4.1 Initial Reactions: Curiosity and Fear

Upon seeing the self‐sampling kit for the first time, participants revealed a range of emotions. One woman initially expressed fear of the unknown but acknowledged the need to overcome it through repeated attempts. Her openness to trying the kit stemmed from a realization that fear is not a solution, emphasizing the importance of overcoming uncertainties:

Firstly, I think of fear. I would be afraid to try and do something […]. It is new to me. It will be hard the first time, but I will not be afraid after trying a few times. Fear is not a solution, so you must overcome all your fears.

Interview 3, 29 years old, uninsured

Participants expressed concerns about waiting for the results and the emotional burden of a potential diagnosis. One young woman expressed her fear of leaving her child behind, showcasing the emotional complexities tied to the anticipation of results and its impact on family responsibilities:

You know what? I might do it, but I would be scared until I got the results. I would like to know what it is like. What is it going to be? I am a young woman; will I leave my child alone?

Interview 1, 42 years old, uninsured

#### 3.4.2 Moving Forward: Gaining Knowledge and Support

For some women, instructions encouraged them to engage in the self‐sampling procedure. They emphasized how detailed guidance, including visual aids, was essential for transitioning from emotional reservations to a more instrumental approach. Participants stressed the importance of explicit guidance in building confidence and self‐efficacy:

Well, I would read it first. That would be the first thing, and the second thing, I would look at the pictures and replicate them step by step.

Interview 6, 28 years old 42, uninsured

Although the instructions helped build confidence, participants still expressed concerns about their anatomical knowledge and the potential for self‐harm. However, they emphasized that following the instructions instilled confidence and made the procedure seem safer. The desire for support from HCPs or family members was a recurring theme, underlining the intricate interplay of emotions and rational considerations:

It is the biggest problem. It is not doing something to myself, not hurting myself because it has this metal tip, and I do not even know if it will do me any good. However, if I follow the instructions, I will make it.

Interview 6, 28 years old, uninsured

The husband, of course. He has been by my side for a long time. He has supported me in all my difficult situations, and of course, I would ask him to help.

Interview 3, 29 years old, uninsured

#### 3.4.3 Embracing Self‐Sampling: Perceived Benefits and Strategies

As participants engaged with the self‐sampling kit, they identified several advantages. These included some perceived benefits such as time efficiency, reduced pressure, no need for appointments to collect the probe, and a diminished sense of shame or embarrassment. Women appreciated the practicality and convenience of self‐sampling, especially its ability to reduce the number of visits to healthcare facilities:

(self‐sampling) is much more comfortable, practical for a woman… and is much handier. It would save many trips in the end.

Interview 11, 33 years old, insured, low SES

Despite the initial concerns, participants were willing to try self‐sampling when provided with explicit guidance. Clear instructions and visual aids were seen as practical strategies to enhance compliance. Even though some uncertainties remained, women felt ready to attempt self‐sampling with proper guidance:

No, I do not think it is that difficult. I am following the pictures or the writing; it would not be a problem.

Interview 11, 33 years old, insured, low SES

## 4 Discussions

The present analysis offers an in‐depth exploration of how vulnerable Romanian women perceive and emotionally respond to CCS and self‐sampling. The findings reveal key factors contributing to low engagement among these populations and underscore the multifaceted challenges that shape women's participation. Four major themes emerged: (a) Risk, fear and other emotional experiences; (b) Women's perception of barriers; (c) Knowledge, personal control and cues to action; and (d) Women's perception of self‐sampling.

Past research shown that logistical barriers to attending CCS significantly affect participation rates [32, 33, 34]. In this study, most women lived in rural, isolated areas with limited or no access to screening services, often requiring long and costly travel. These logistical challenges, including extended travel times, poor accessibility and financial constraints, were compounded by poverty which emerged as a persistent deterrent to seeking medical care. The combined impact of being uninsured and having a low SES further discouraged participation in screening consistent with previous findings linking financial hardship to reduced healthcare access [35]. In the present analysis, many participants noted that prioritizing family needs and daily expenses over personal health led to symptom‐driven visits rather than preventive care. This also contributed to difficulties accessing the referral system, contributing to longer waiting times [36, 37]. This perspective underscores the need for a shift from symptom‐based care to preventive health practices, emphasizing the importance of regular check‐ups as proactive measures for maintaining overall health and well‐being.

Beyond logistical and financial deterrents, living in remote areas often correlated with limited health literacy, particularly regarding CC or CCS, which further exacerbates the existing barriers. Earlier published research unveiled an intricate relationship between knowledge and screening attendance [38]. A lack of knowledge often breeds fear, with some participants expressing anxiety about receiving a positive result. The term ‘cancer’ was rarely mentioned explicitly; instead, a few women conveyed a fatalistic attitude towards their expected results and the impact of potentially leaving loved ones behind. Earlier studies have shown that the emotional burden of anticipating a positive result can deter women from participating in CCS [32, 37, 39], especially when worrying about coping [40]. The fear of a cancer diagnosis was a major factor contributing to low CCS participation.

Emotional responses, particularly personal risk assessment emerged as a central theme, revealing diverse perceptions of susceptibility to CC. Limited knowledge about CC and CCS often distorted women's risk evaluations, leading to feelings of helplessness and low self‐efficacy. Some participants viewed factors such as faith in divinity or age were often perceived as protective against the risk of illness and suffering from CC. The combination of limited knowledge and fear of a positive result became a significant barrier to CCS participation. In line with present findings, published research uncovered anticipated regret and fear associated with a potential CC diagnosis to further diminish enthusiasm for screening participation [16, 35, 36, 40].

Cultural beliefs played a significant role in shaping women's attitudes towards CCS and self‐sampling. For instance, several participants expressed a firm reliance on divine protection, suggesting that faith in God reduced their perceived vulnerability to illness. This belief may contribute to a lower perceived need for preventive screening. Additionally, age‐related modesty and societal expectations around body exposure were frequently cited as barriers. These gendered norms around bodily privacy and shame were deeply embedded and discouraged participation in gynaecological exams. Furthermore, stigma surrounding cancer itself was evident, with one participant avoiding discussion of risk altogether. These examples highlight the need for culturally sensitive messaging and interventions that acknowledge and address such beliefs directly and resonate with an extensive body of research that has revealed a cultural aversion to preventive behaviours among certain ethnic groups [41]. Examples include a study in the United Kingdom describing how emotional barriers, such as fear, embarrassment and shame, were more prominent among Asian women, exacerbated by stigma associated with the view that CC is linked to sexual activity outside marriage [36]. A systematic review of CCS uptake among immigrant Muslim women highlighted the importance of modesty, virginity and gender norms as significant barriers to screening, along with religious views that illness is a punishment from God or that health outcomes are predetermined by divine will [42].

These findings reveal a vicious cycle affecting underserved vulnerable women in remote areas: limited access to information reduces their likelihood of engaging in CCS, while long travel distances and financial hardship further hinder participation. This underscores the urgent need for improved health education and targeted programmes in remote regions to address these barriers and promote equitable access to screening services.

Shame or embarrassment associated with gynaecological examinations also emerged as an influential factor, driving women towards self‐sampling, aligning with previous research [15, 40, 43, 44]. Past experiences with HCPs shaped emotional responses and influenced future healthcare engagement with healthcare services, including screening. Negative healthcare experiences often discouraged women from seeking care or participating in preventive measures. In contrast, positive interactions with HCPs, marked by effective communication and clear treatment pathways, can boost women's willingness to participate in screening programmes, enhancing their self‐efficacy and perception of the benefits [45, 46]. Mistrust in the healthcare system and professionals was also noted among vulnerable women, extending beyond CCS to include vaccinations. This highlights the need for more accurate information and trust‐building relationships between HCPs and patients [45, 46]. Previous research has similarly identified widespread mistrust among vulnerable populations, including refugees in Romania [47].

Self‐sampling has proven effective in addressing some of the negative emotions associated with CCS. However, existing literature lacks a detailed analysis of its acceptability among vulnerable Romanian women. This study used semi‐structured interviews combined with the Think Aloud protocol to gain deeper insight into participants' perceptions and experiences, giving voice to underserved populations, an essential step in designing tailored interventions. Insights from the Think Aloud Protocol uncovered women's emotional reactions and cognitive responses to self‐sampling. Initial responses ranged from fear and apprehension to increased willingness after becoming familiar with the kit. Fear was a recurring theme, encompassing anxiety about the procedure, the anticipation of results and the emotional weight of a potential diagnosis. Clear instructions played a crucial in easing these concerns, fostering confidence and self‐efficacy. Despite this, some women remained uncertain about their anatomical knowledge and feared causing harm, highlighting ongoing challenges in the problem‐solving process. Many expressed a need for support from HCPs or family members, illustrating the intricate interplay between emotional, rational and informational factors in their decision to use self‐sampling [33, 39, 48, 49, 50, 51].

### 4.1 Study Limitations

A limitation of the current analysis is represented by the relatively small sample size as only 18 participants completed the interviews. This was partly due to the chain‐referral sampling method used, which limits researchers' control over recruitment beyond the initial participants. Since recruitment depends on participants inviting others, there is no guarantee that new individuals will respond positively, often resulting in extended recruitment periods, as observed in this study. Despite these challenges, the recruitment strategy had notable strengths. It successfully reached typically underserved populations and, by involving members of their own communities, helped encourage sceptical women to participate. This peer‐driven approach was instrumental in building trust and facilitating engagement.

Another limitation of this study was the use of both face‐to‐face and telephone interviews depending on logistical feasibility, comfort level and availability. Given the sensitive and potentially stigmatizing nature of CCS, offering both modalities allowed participants to choose the format that felt safest and most convenient for them, thereby supporting ethical inclusivity and participant autonomy. While this approach was primarily pragmatic, it allowed researchers to involve more vulnerable women and have more flexibility regarding recruitment and geographic distance. This approach enabled the investigators to reach a diverse range of participants who might otherwise have been excluded. Face‐to‐face interviews facilitate richer emotional expression through non‐verbal cues and deepen rapport. On the other hand, telephone interviews can reduce visual contact but sometimes promote greater openness when discussing intimate or sensitive experiences. They also helped broaden access by removing transportation barriers and reducing the financial strain associated with travel to in‐person interviews. Further, they offered participants a greater sense of anonymity, which encouraged more open emotional expression. However, this method limited the interviewer's ability to observe non‐verbal cues and made it more challenging to build rapport, an issue noted in previous research [52]. To ensure consistency, the same semi‐structured interview guide was used across all interviews, and all sessions were conducted by the same trained interviewer using a reflective, empathic approach. Field notes were taken after each interview to document tone, pauses and contextual impressions. Few studies have highlighted that conducting telephone interviews adequately can elicit data of comparable richness to face‐to‐face methods, particularly in studies addressing sensitive or health‐related topics [53, 54]. During analysis, no meaningful differences were observed in the thematic content or emotional depth of responses between face‐to‐face and telephone interviews. In fact, some participants appeared to speak more freely by telephone, possibly due to the greater sense of anonymity it provided. Overall, both modalities yielded rich and comparable data that contributed equally to the understanding of participants' emotional and cognitive perceptions of CCS and self‐sampling for HPV.

A limiting factor to the interpretability of the results was the underrepresentation of certain marginalized groups and the small number of participants involved in the Think Aloud protocol. Groups such as women experiencing addiction, chronic mental illness, homelessness, incarceration or sexual and physical violence, who face significant barriers to healthcare, were not adequately represented. Additionally, the study was limited to participants from only two counties in Romania, which may restrict the diversity of experiences with HCPs, as well as the range of barriers and emotional responses to CCS and self‐sampling. The homogeneity of the sample can be attributed to the chain‐referral sampling method, where participants tend to recruit others from similar backgrounds and communities. Nonetheless, given that over 40% of Romania's population lives in rural areas, many of which are medically underserved, the selected participants remain highly relevant for exploring the experiences of vulnerable Romanian women [18]. It is important to acknowledge, however, that the findings may not be generalizable to other vulnerable groups, such as urban poor populations, migrant communities, LGBTQ+ individuals and women with disabilities. These groups may face distinct barriers, including different forms of stigma, healthcare access issues or cultural beliefs that influence screening behaviours.

Despite these limitations, the study offers valuable insights that can inform future research and intervention design for other underserved populations, helping ensure that programmes are inclusive and responsive to diverse social and geographic contexts.

### 4.2 Future Directions and Implications

This study highlights a complex synergistic vicious circle that deter vulnerable Romanian women from attending CCS. Living in remote, isolated areas, combined with limited education and awareness, and financial strain, creates barriers that go beyond individual or systemic challenges. These factors heighten perceived risk and promote symptom‐driven healthcare visits over regular preventive screenings. Addressing these issues requires coordinated action at multiple levels, including the development of inclusive health policies, targeted education and enhanced access to services, to ensure universal and equitable participation in CCS.

First, low awareness and limited health education must be addressed by specific targeted educational interventions in vulnerable communities. Previous research underscored the need for targeted educational interventions [32, 33, 34]. Tailored educational campaigns might improve addressability while raising awareness and knowledge. School‐based initiatives have shown to improve vaccination rates among vulnerable Romanian children [55]. Based on translational principles, school‐based educational campaigns might improve screening rates, although results might be observed on the longer term. Published work has also suggested the role of engaging members of their own community to deliver interventions among vulnerable populations and deemed the procedure as effective [56]. This can also boost confidence while lowering potential cultural aversion to screening procedures.

Second, logistical barriers must be addressed in these underserved communities. Mobile units not only enhance awareness, but mitigate the barriers faced by communities living in isolated areas. These units help alleviate the financial burden of long commutes, ease the navigating process and reach underserved communities. Encouragement from HCPs and family members has been previously identified as a key factor in boosting women's intention to participate in screening [35, 36]. Strategies must be deployed to engage HCPs in screening campaigns. Family doctors and General practitioners in isolated communities can prompt vulnerable women to engage in preventive behaviours and perhaps can also actively screen women. In addition, these individuals can contribute to improving education.

Third, the lack of financial resources must be mitigated by offering reimbursed universal screening. The Romanian health policy states that even uninsured populations benefit from free cancer treatments and become automatically insured. However, it is important to recognize the financial constraints not only at the individual level but also within the healthcare system. Future research should explore the economic implications of offering free screening alongside free treatment for CC to support sustainable and equitable policy development.

Lastly, the study's practical recommendations must be incorporated into policy and practice to increase CCS participation, particularly among vulnerable populations. This involves moving beyond conventional healthcare approaches by establishing specialized screening centres, deploying mobile units for outreach, creating clear and accessible instructional materials and offering adequate support during self‐sampling. This becomes especially relevant as Romania has recently adopted a National Plan for Combating Cancer. As part of recent endeavours, the plan strives to kickstart and optimize nation‐based screening programmes for major cancers including CC. Vulnerable populations must be taken into consideration as it constitutes significant proportion of the targeted population

Future research must thoroughly explore the specific needs and priorities of these groups needs, to develop tailored solutions and clinically relevant interventions. Mitigating the apprehension associated with a positive result while simultaneously acquainting women with self‐sampling kits, thereby fostering proactive engagement in preventive care. Mixed‐methods research, which combines qualitative and quantitative data, can provide a deeper and more comprehensive understanding of the barriers and facilitators to CCS and self‐sampling. Longitudinal studies should be priority should be given to longitudinal designs, that allow traking changes over time in trust towards HCPs perceived risk, emotional responses and sustained use of self‐sampling. Evaluating the long‐term impact of targeted educational interventions on health literacy, screening intentions and participation rates will provide valuable insights for designing effective, inclusive strategies. Such studies should include changes in trust towards HCPs and institutions, shifts in perceived risk and emotional responses to CCS, and the uptake and sustained use of self‐sampling methods.

Finally, regional initiatives should consider introducing self‐sampling in isolated, vulnerable communities, particularly among ethnic minorities in Romania, and evaluate its effect on improving access and participation.

## 5 Conclusion

This study identified the multifaceted challenges hindering vulnerable Romanian women's engagement in CCS, including logistical barriers, financial constraints and limited access to information. These interconnected factors underscore the need for targeted interventions such as medical education programmes and tailored screening campaigns including mobile screening units.

The Think Aloud Protocol offered valuable granular insights into vulnerable women's initial apprehension and subsequent growing confidence with self‐sampling. Clear instructions were essential in reducing fear and building self‐efficacy, with support from HCPs and family members playing a crucial role. To improve access, establishing specialized screening centres and support systems is recommended to address both logistical and emotional barriers. Self‐sampling may serve as an effective complementary tool to increase participation, particularly in underserved areas. Shifting health policy towards inclusive tactics that prioritize vulnerable populations is essential for improving health outcomes and ensuring equitable access to preventive care

## Author Contributions

**Nicoleta‐Monica Pașca:** investigation, writing – original draft, methodology, writing – review and editing, formal analysis, data curation. **Diana Tăut:** conceptualization, writing – original draft, methodology, investigation, writing – review and editing, formal analysis, data curation. **Rachel Greenley:** investigation, conceptualization, writing – review and editing, formal analysis, data curation. **Pia Kirkegaard:** conceptualization, writing – review and editing. **Rikke Buus Bøje:** conceptualization, writing – review and editing. **Martin McKee:** writing – review and editing. **Marc Bardou:** conceptualization, writing – review and editing. **Adriana‐Smaranda Băban:** conceptualization, writing – original draft, investigation, methodology, writing – review and editing, formal analysis, supervision. **CBIG‐SCREEN Consortium:** conceptualization, funding.

## Ethics Statement

The study was approved by the Ethical Committee of Babes‐Bolyai University, Cluj‐Napoca, Reference Number 557/29.11.2022.

## Conflicts of Interest

The authors declare no conflicts of interest.

## Supporting information

Interview guide including self‐sampling questions.

## Data Availability

Data available on request.
