# Supplementary material for: Exploring Emotional and Cognitive Perceptions of Cervical Cancer Screening and Self‐Sampling Among Vulnerable Romanian Women: A Qualitative Study
Source: Health Expect. 2025 Nov 30;28(6):e70485. doi: 10.1111/hex.70485 (PMC12665870; doi:10.1111/hex.70485)
Supplement: Supplementary file 1 — Interview guide including self‐sampling questions. [file HEX-28-e70485-s001.docx]

| Overview image of the participant   1. **For start, I would like to know you better. Can you tell me a bit about yourself?** | ANCHORS (make sure the following info is touched upon):  *Where do you live*  *where do you come from?*  *Your studies/qualifications?*  *Work?*  *Relationship/partnership?*  *Children?*  *What is your current life situation?* |
| --- | --- |
| Experiences and perceptions of the healthcare system   1. **What are your views and opinions on your health and healthcare in general?** 2. **How would you describe your experiences and encounters with healthcare?** 3. **If you were seen by doctors/nurses how would you describe your experience? What made your experience like that?** | ANCHORS:  *What can you tell me about your health insurance status?*  *What are the health issues that you’ve dealt with?*  *How did you deal with them?*  *If you dealt with these issues in another way (she didn’t see a doctor), what were the reasons for this?* |
| Knowledge about & experience with CC   1. **As you were already informed, this project is about ways to improve cervical cancer screening. In your role as a potential beneficiary/patient, I’m curious to know what sort of information you have about cervical cancer.** 2. **What are the things that make you {use participants' words in her answer to question 7} for CC in the future?**   *TIP: use participants’ own words in her answer to question 7. She might talk about “high/medium/low risk” or “vulnerability” or other term to describe her likelihood of developing CC in the future.*   1. **How do you perceive your risk at getting CC? Why is that?** | ANCHORS  What is your experience with CC?  *Do you know anyone who suffered from CC?*  *Where did you hear about it?*  *What did you hear about it?* How are your thoughts/emotions about CC and your own risk of getting it |
| Screening/Prevention of CC   1. **We discussed about CC which, naturally, makes us touch upon a related topic: What about cervical cancer prevention, what has been your experience with that?**   IF the woman doesn’t mention Pap Smear or HPV and they never heard of them, we briefly explain them (Interviewer is expected to have basic knowledge about CCS and HPV)   1. **What is your experience with CCS?** 2. **Pap Smear/HPV *{insert country situation here*} is a free service for women over *{insert country situation here*}. However, the coverage is not 100%. For instance in {*Country*}, it is around {*insert country statistics here*}. Why do you think we have this situation? What would be possible**   **explanations?**   1. **Can you think any barriers for you to participate in CCS?** 2. **In terms of access** 3. **In terms of the procedure** 4. **In terms of follow up** 5. **What would have to happen to make sure you have a CCS test?** 6. **What about the women you know? How likely would it be for them to get screening? Why?** 7. **Suppose you are heads-on convinced that CCS is something that you will have to do for your health. In what scenario/what would have to happen for you to change your mind and NOT perform CCS?** | **ANCHORING questions:**  *Did you have any information about* *CCS/HPV vaccination?*  *From where did you get the information?*  *What were you told?*  ANCHORS:  Is it something you’ve already done? When?  How was it for you?  Do you plan to keep on doing it whenever it is recommended?  *If not: Is it something you would do? Why/why not?*  ANCHORS:  *What are your fears/worries regarding CCS? What about other women’s concerns?* |
| Self- sampling | **Anchoring questions** |
| **As part of this project we will investigate if self-sampling is a feasible way to make more women participate in CCS. Show a self- sampling kit and explain how it is done concretely (including the procedure of receiving, returning and getting test result).**  **18. What do you think of such a screening system?**  **19. Would you do it? (explore their concerns if any)**  **20. Do you think other women would do it? (those who don't currently screen for example)**  **21. How would you prefer to receive / return the kit? (post, pharmacy, doctor, do it yourself in a lab...**) | *Do you have any concerns about this method?*  *Would you feel safe do to it on your own?*  *Would you prefer to have support from someone (whom?)* |
| Closure  **22. Thank you so much for openness to share your story and experiences with me. Women are experts in what is/what is not a sensible, feasible, right approach to CCS, since we are the beneficiaries of it. Although there is a lot of information I’ve heard from you and a lot of very useful input, I’m not sure whether you had the chance to tell your full story and opinion on CC and CCS. Are there any other things related to CC and CCS that we should explore?** |  |
